# Exploring the relationship between patellofemoral instability and bone morphology: discoveries and challenges

**DOI:** 10.1590/0100-3984.2023.56.6e4-en

**Published:** 2023

**Authors:** André Yui Aihara

**Affiliations:** 1 Adjunct Professor in the Department of Diagnostic Imaging at the Escola Paulista de Medicina da Universidade Federal de São Paulo (EPM-Unifesp), São Paulo, SP, Brazil. Email: andre.aihara@unifesp.br

Monitoring changes in bone development is crucial for identifying problems early, enabling the necessary interventions and treatments to ensure healthy bone growth and prevent future complications such as the early development of osteoarthritis^(1)^.

Imaging examinations, including X-rays, magnetic resonance imaging scans, and computed tomography scans, play an essential role in the evaluation of abnormalities in bone development. Such examinations provide detailed information about bone morphology, allowing accurate diagnoses, as well as improving treatment planning, the monitoring of the progression of the condition, and the determination of the effectiveness of treatments^(2,3)^.

Patellofemoral instability is common in young patients and is a common indication for imaging evaluation. Bone abnormalities in the patella, femoral trochlea, and tibia are crucial factors contributing to the condition. In the management of cases of patellofemoral instability, especially when considering a surgical approach, physicians often request imaging examinations to measure and quantify these bone changes^(4,5)^, through measurement of the height, lateralization, and inclination of the patella; determination of the degree of trochlear dysplasia; and measurement of the tibial tuberosity–trochlear groove distance. It has been suggested that other imaging findings, not restricted to the knee, such as femoral torsion, tibial torsion, and frontal mechanical axis deviation of the lower limb, are potential factors contributing to patellofemoral instability^(6)^.

The study conducted by Jacob et al.^(7)^ and published in this issue of **Radiologia Brasileira** explores a little-studied aspect of patellofemoral instability: its relationship with the medial femoral condyle. The authors performed a morphological analysis of the femoral condyles on MRI scans of adolescents and young adults, divided into a group with trochlear dysplasia and another (control) group without signs of patellofemoral instability. The most relevant finding was hypoplasia of the medial femoral condyle in young patients with trochlear dysplasia in comparison with the controls. However, their study has some limitations, such as the relatively small sample size and the heterogeneity among the individuals analyzed regarding the stage of skeletal development. Studies with larger samples could consolidate this evidence and analyze differences between genders and among ethnic groups. Investigations of populations undergoing skeletal maturation could clarify when and how condylar hypoplasia manifests. Another line of research is the possible interrelationships among patellofemoral instability, hypoplasia of the medial femoral condyle, and changes in the femorotibial compartments. Finally, it is essential to understand the clinical relevance of such hypoplasia in patellofemoral instability.

Imaging examinations play a crucial role in the evaluation of patients with pain in the anterior region of the knee^(2,3,4,5,6)^, and the Jacob et al.^(7)^ study highlights a little-explored aspect of patellofemoral dysfunction: hypoplasia of the medial femoral condyle. Their findings underscore the need for further research to expand our understanding of patellofemoral instability and to improve targeted care for patients with this condition.
